# Clinical Trial Eligibility Criteria and Recently Approved Cancer Therapies for Patients With Brain Metastases

**DOI:** 10.3389/fonc.2021.780379

**Published:** 2022-01-03

**Authors:** Aaron C. Tan, Drexell H. Boggs, Eudocia Q. Lee, Michelle M. Kim, Minesh P. Mehta, Mustafa Khasraw

**Affiliations:** ^1^ Division of Medical Oncology, National Cancer Centre Singapore, Singapore, Singapore; ^2^ Duke-NUS Medical School, National University of Singapore, Singapore, Singapore; ^3^ Department of Radiation Oncology, University of Alabama at Birmingham School of Medicine, Birmingham, AL, United States; ^4^ Center for Neuro-Oncology, Dana-Farber Cancer Institute, Boston, MA, United States; ^5^ Department of Radiation Oncology, University of Michigan, Ann Arbor, MI, United States; ^6^ Department of Radiation Oncology, Miami Cancer Institute, Baptist Health South Florida, Miami, FL, United States; ^7^ Duke Cancer Institute, Duke University, Durham, NC, United States

**Keywords:** brain metastases, trial eligibility, intracranial efficacy, novel therapeutic agents, CNS metastases

## Abstract

Brain metastases cause significant morbidity and mortality in patients with advanced cancer. In the era of precision oncology and immunotherapy, there are rapidly evolving systemic treatment options. These novel therapies may have variable intracranial efficacy, and patients with brain metastases remain a population of special interest. Typically, only patients with stable, asymptomatic and/or treated brain metastases are enrolled in clinical trials, or may be excluded altogether, particularly in the setting of leptomeningeal carcinomatosis. Consequently, this leads to significant concerns on the external validity of clinical trial evidence to real-world clinical practice. Here we describe the current trends in cancer clinical trial eligibility for patients with brain metastases in both early and late phase trials, with a focus on targeted and immunotherapies. We evaluate recent newly FDA approved therapies and the clinical trial evidence base leading to approval. This includes analysis of inclusion and exclusion criteria, requirements for baseline screening for brain metastases, surveillance cerebral imaging and incorporation of trial endpoints for patients with brain metastases. Finally, the use of alternative sources of data such as real-world evidence with registries and collaborative studies will be discussed.

## Introduction

Brain or central nervous system (CNS) metastases remain a significant cause of morbidity and mortality in patients with advanced cancers (1). The incidence of brain metastases may be increasing, in part due to greater detection through routine cerebral imaging and more effective systemic therapies allowing later manifestations of the disease to occur (2). Particularly in the era of precision oncology and immunotherapy, there are rapidly evolving systemic treatment options for many cancers. These novel therapies may have variable intracranial efficacy, and patients with brain metastases remain a population of special interest (3). Typically, only patients with stable, asymptomatic, and/or treated brain metastases are enrolled in clinical trials, or may be excluded altogether, particularly in the setting of leptomeningeal carcinomatosis. Consequently, this leads to significant concerns on the external validity of clinical trial evidence to real-world clinical practice (4).

In this review, we describe the current trends in cancer clinical trial eligibility for patients with brain metastases in both early and late phase trials, with a focus on recently approved targeted and immunotherapies. The United States (US) Food and Drug Administration (FDA) approved therapies from 2018-2020 and the clinical trial evidence base leading to approval are evaluated. Key recommendations previously published by the American Society of Clinical Oncology (ASCO)–Friends of Cancer Research (FCR) Brain Metastases Working Group for the inclusion of patients with brain metastases in clinical trials to improve generalizability of trial evidence are considered (5). This includes an analysis of inclusion and exclusion criteria, requirements for baseline screening for brain metastases, surveillance cerebral imaging and incorporation of trial endpoints for patients with brain metastases. Finally, the use of alternative sources of evidence such as real-world evidence with registries and collaborative studies are discussed.

## Analysis of Recently FDA Approved Cancer Therapies

**Figure 1:**
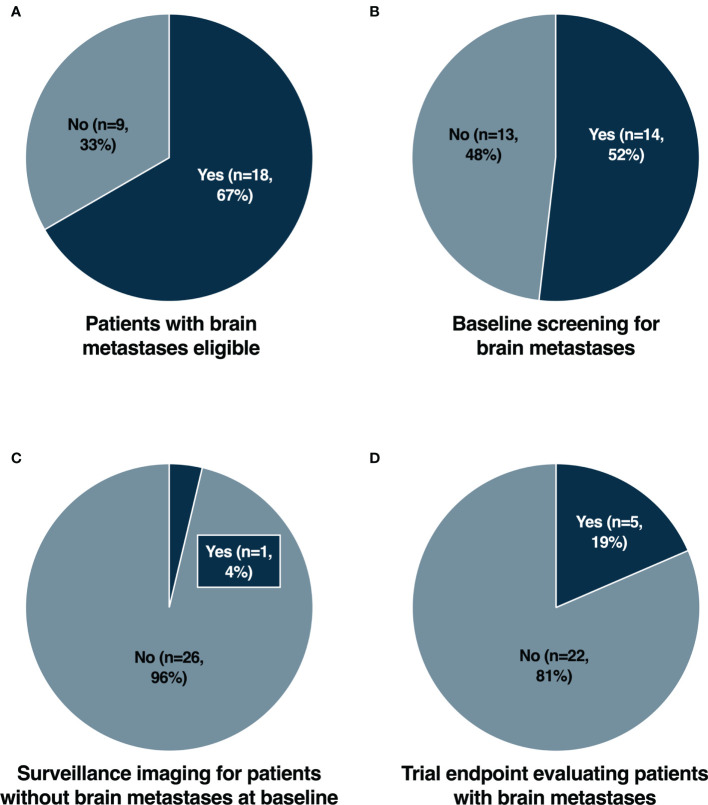
Eligibility for patients with brain metastases **(A)**, baseline screening for brain metastases **(B)**, surveillance imaging for patients without brain metastases at baseline **(C)** and trial endpoint evaluating patients with brain metastases **(D)** in registrational trials for newly FDA approved cancer therapies from 2018-2020.

We conducted an analysis of newly FDA approved cancer therapies from 2018-2020 (6–8) as shown in **Supplementary Table 1**. For each cancer therapy, the registrational trial leading to regulatory approval was evaluated. The characteristics of the registrational trials are summarized in **Supplementary Table 2**. Trials conducted in the metastatic or late-stage cancer setting were initially assessed for eligibility for patients with brain metastases. Of 27 trials, 18 (67%) allowed enrollment of patients with stable and asymptomatic brain metastases (**Figure 1A**). Baseline screening for brain metastases with CT or MR imaging was required in 14/27 (52%) trials (**Figure 1B**). Surveillance cerebral imaging in patients without brain metastases at baseline was required in only 1/27 (4%) trials (**Figure 1C**). A prespecified trial endpoint evaluating patients with brain metastases was incorporated in 5/27 (19%) trials (**Figure 1D**).

## Eligibility of Patients With Brain Metastases

Patients with brain metastases have historically been excluded from clinical trials due to concerns relating to overall greater risks of toxicity and poorer survival outcomes. With improved local therapeutic options for brain metastases and greater survival outcomes, assessment of intracranial efficacy and toxicity is becoming ever more important. The potential lack of blood-brain barrier (BBB) penetration for novel therapies is also often cited as a rationale for exclusion. However, for established brain metastases, tumors compromise the integrity of the BBB acquiring neovasculature and a resulting blood-tumor barrier (9). Consequently, increasingly there are trends to include patients with stable, treated and/or asymptomatic brain metastases (10). This approach would improve the generalizability of trial results, particularly in cancer types with a high prevalence of brain metastases. Nevertheless, from our analysis (**Figure 1A**), we found that a significant proportion of recently approved cancer therapies still exclude patients with brain metastases from the initial registrational trials. This included trials in tumor types with a low overall prevalence of brain metastases, such as NAVIGATOR (avapritinib) (11) and INVICTUS (ripretinib) (12) for gastrointestinal stromal tumors (GIST) and Study EZH-202 (tazemetostat) (13) for epithelioid sarcomas. In addition, there were cases in which CNS disease may represent a distinct clinical entity such as CNS lymphoma (14, 15). However, even in cancers with a high prevalence of brain metastases, there were examples of trials which completely excluded patients with brain metastases. Notably this included small cell lung cancer (SCLC) with Study B-005 (lurbinectedin) (16), *EGFR* mutated non-small cell lung cancer (NSCLC) with ARCHER 1050 (dacomitinib) (17) and triple negative breast cancer (TNBC) with IMMU-132-01 (sacituzumab govitecan) (18). The lack of evidence for intracranial efficacy of agents that have received regulatory approval represents a significant limitation for these compounds (19, 20). Particularly for *EGFR* mutated NSCLC and TNBC where the prevalence of brain metastases may be as high as 32% and 46% respectively (21–23). Whilst patients with brain metastases may have been subsequently allowed in larger phase 3 trials such as for lurbinectedin (24) and sacituzumab govitecan (25), the initial exclusion also necessitated the initiation of further trials to generate data for this important patient subpopulation (26, 27). Furthermore, the rationale for excluding patients with brain metastases was not elaborated upon in the primary publications. The lack of efficacy and safety data from early phase trials may have been a contributory factor. However, lurbinectedin (28, 29) and dacomitinib (30) for example had allowed patients with non-progressive or treated/stable brain metastases in earlier trials.

## Baseline Screening for Brain Metastases and Surveillance Cerebral Imaging

Screening for brain metastases at baseline is a common cause of screen failure, particularly in early phase clinical trials (31). Consequently, unless mandated this may lead to hesitancy from clinicians to perform cerebral imaging for risk of jeopardizing a patient’s eligibility for trials (5). As trials increasingly allow patients with stable and treated brain metastases however, more completely characterizing the intracranial efficacy of novel therapies also becomes of heightened importance. From our analysis, nearly half (48%) of trials did not require mandatory cerebral imaging at baseline (**Figure 1B**), and only one (4%) trial required surveillance imaging for patients without brain metastases at baseline (**Figure 1C**). For many trials, cerebral imaging during screening was at least required with known or suspected brain metastases. However, there is increasing evidence supporting routine standard of care screening for brain metastases in many cancers, such as advanced breast cancer, melanoma and NSCLC, both at diagnosis and after initiation of palliative systemic therapy (32). Despite this, there remained trials such as SOLAR-1 (alpelisib) (33), SOPHIA (margetuximab) (34), ARROW (pralsetinib) (35) and LIBRETTO-001 (selpercatinib) (36), that did not mandate baseline cerebral imaging. The NAVIGATOR trial (11), a phase 1 study of avapritinib which included a dose expansion cohort for *PDGFRA*-mutated GIST patients, was the only study with regular surveillance cerebral imaging. However, this was performed due to safety concerns regarding an increased risk of intracranial bleeding, rather than generating data elucidating intracranial efficacy. In addition to intracranial response rates, measures of intracranial efficacy such as time to CNS progression, are also increasingly reported. Therefore, the role for routine surveillance cerebral imaging may become important in cancers with a propensity for the development of brain metastases.

## Protocol Specific Management of Intracranial Progression

The treatment paradigm for brain metastases includes a multimodality approach including surgery, radiation therapy and systemic therapy (3). Consequently, for patients that experience isolated intracranial progression, local therapeutic approaches whilst continuing systemic therapy beyond progression represents a commonly adopted clinical approach. For clinical trials, specific protocol guidance in such instances is crucial to safely and effectively treat progressive brain metastases whilst collecting adequate data for CNS outcomes. For example, the COLUMBUS trial evaluating encorafenib plus binimetinib in patients with *BRAF* mutated melanoma (37), specified the potential for dosing beyond progression for new brain metastases treatable with stereotactic radiotherapy or surgery but not requiring whole brain radiotherapy. However, for a large majority of trials that allowed treatment beyond progression, there was no specific protocol guidance for the management of intracranial progression.

## Incorporation of Trial Endpoints for Patients With Brain Metastases

With the improving intracranial efficacy of many novel targeted and immunotherapies compared with traditional chemotherapy, the prospective evaluation of CNS outcomes with prespecified endpoints is also becoming paramount. In our analysis, only a small number of registrational trials prespecified a trial endpoint evaluating patients with brain metastases (**Figure 1D**). This included Study B7461001 of lorlatinib for patients with *ALK* rearranged NSCLC (38), which included intracranial objective response rates as a co-primary endpoint. The remaining trials incorporated secondary endpoints assessing intracranial response and/or time to intracranial progression, including trials such as ARROW (pralsetinib) (35) and LIBRETTO-001 (selpercatinib) (36) for *RET* rearranged NSCLC, HER2CLIMB (tucatinib) (39) for *HER2* positive breast cancer and ALKA/STARTRK-1/STARTRK-2 (entrectinib) (40, 41) for *NTRK* rearranged solid tumors and *ROS1* rearranged NSCLC. *Post hoc* analyses describing outcomes for patients with brain metastases have subsequently been reported from many of the other registrational trials. However, without prospective plans to evaluate CNS response and progression, results may be more exploratory. For example, in the DESTINY-Breast01 trial of trastuzumab deruxtecan in *HER2* positive breast cancer (42), there was a cap of patients with brain metastases allowed for enrolment. Intracranial efficacy from this trial has been shown to be promising (43), however further prospective trials for patients with brain metastases have been initiated to better characterize the CNS activity (27).

## Alternative Sources of Data Such as Real-World Evidence

Despite increasing trends to include patients with brain metastases in clinical trials there remains subpopulations of patients that often remain excluded. This includes patients with leptomeningeal disease and symptomatic or active (new and/or progressive) brain metastases. Safety considerations or unsupportive pre-clinical evidence are potential reasons where exclusion from trials may still be appropriate (5). Therefore, there is heightened need for alternative sources of evidence in such populations, for which real-world evidence may provide an opportunity. Data sharing and collaboration through multi-center registries and trial networks are potential avenues to integrate supportive real-world evidence to clinical trial data (44). Particularly given the relative rarity of these subpopulations, pooled analyses may represent efficient methods of generating high quality prospective data. The Brain Metastases in Breast Cancer Network Germany is one such example (45), however future efforts need to be driven by both academia and industry. With rapid development of targeted and immunotherapies with unique mechanisms of action, greater reverse translation of our biological understanding of novel compounds from real-world evidence and clinical trial data to inform pre-clinical models and drug discovery pipelines is also paramount (46).

## Discussion

In our analysis, a significant proportion of registrational trials for new recently FDA approved cancer therapies allowed patients with brain metastases. However, there remained prominent examples of trials which excluded even stable or asymptomatic brain metastases. These trials may have been conducted in tumor types with extremely rare incidence of brain metastases, such as GIST (47). Nevertheless, the relatively rarity of brain metastases in these tumor types may not represent sufficient rationale for automatic exclusion from clinical trials – particularly when baseline screening for brain metastases is mandated. Whilst most trials also required baseline screening for brain metastases, only a minority of trials required surveillance CNS imaging for patients without baseline brain metastases, had protocol specified guidelines for intracranial progression, or incorporated a trial endpoint for patients with brain metastases. This highlights important areas in which we can improve our understanding of the intracranial activity of novel therapies from clinical trials (**Table 1**).

**Table 1:** Recommendations to improve the clinical trial eligibility criteria and evaluation of patients with brain metastases.

Recommendations	
Eligibility of patients with brain metastases	Routine inclusion of patients with treated and/or stable brain or central nervous system (CNS) metastases Strong consideration to include patients with active brain metastases (or enrolled as a defined subgroup) if there is sufficient scientific rationale for the investigational drug
Baseline screening for brain metastases and surveillance cerebral imaging	Routine baseline screening for brain metastases if incidence of brain metastases is high and/or intracranial efficacy is a predefined endpoint Regular surveillance CNS imaging if the risk of developing brain metastases is high and/or intracranial efficacy is a predefined endpoint
Protocol specific management of intracranial progression	Prospective guidelines specifying management of new or progressive brain metastases, including the allowance for local therapies and continuation of investigational drug
Incorporation of trial endpoints for patients with brain metastases	Incorporation of prespecified trial endpoints for CNS-related efficacy outcomes if incidence or risk of developing brain metastases is high and/or the investigational drug has strong scientific rationale for intracranial efficacy

Recently, the FDA released specific guidance for industry, outlining recommendations for the inclusion of patients with brain metastases (48). It is emphasized that patients with active brain metastases or leptomeningeal disease should not be automatically excluded from trials. Eligibility in early drug development trials to inform criteria for later-phase trials, mitigation of uncertainties with separate subgroups within trials and the importance of CNS imaging at regular intervals are other key recommendations. Nevertheless, deeper considerations of risk benefit ratio with regards to cancer type, disease stage, known pre-clinical data and drug safety profile are all clearly influential in the development of clinical trial eligibility criteria and protocols. For novel therapies with unique mechanisms of action, such as newer immunotherapies, the potential for distinct toxicity profiles or adverse events due to CNS tumor inflammation and/or psuedoprogression is a relevant concern (49). However, from trials to date of immune checkpoint inhibitors in patients with brain metastases, the rates of toxicities and neurologic adverse events do not appear significantly different, and deaths due to neurologic complications remain rare (50). A greater molecular understanding of the development and progression of brain metastases within the unique brain microenvironment is also driving advances with more precise approaches for local and systemic therapies (51). This has broader implications for the data generated from the inclusion of patients with brain metastases on trials evaluating novel compounds, where combination approaches may enhance the intracranial efficacy. Costs and impacts on trial efficiency however, remain other practical considerations (5). Regular surveillance CNS imaging for example, particularly with MRI may be burdensome for patients with tumor types with low prevalence of brain metastases. Our analysis must therefore also be viewed in the context of a markedly heterogenous collation of therapies and registrational trials. In addition, given the length of time required for drug development from early to late phase trials and regulatory approval, the trials in our analysis may not be wholly representative of more contemporary practices in trial protocol design. Ultimately however, the drug development landscape is rapidly evolving with an increasing incidence of accelerated approvals from early phase trials. Consequently, the critical evaluation of clinical trial evidence and its generalizability across the patient population is of heightened relevance.

With an increasing prevalence of patients with brain metastases, understanding the intracranial efficacy of novel therapies is crucial. Expanding the eligibility of patients with brain metastases in registrational trials, or the incorporation of procedures or endpoints in trial design will generate important high-quality data in this patient population with significant unmet need. This will enhance our ability to integrate systemic therapies in the multimodality treatment of patients with brain metastases.

## Authors Contributions

AT and MK contributed to conception and design of the study. All authors contributed to manuscript preparation, read, and approved the submitted version.

## Conflict of Interest

AT reports consultant or advisory roles for Amgen; outside of the submitted work. DB reports honoraria from Varian Medical Systems; and research funding from Varian Medical Systems and Novocure; outside of the submitted work. EL reports honoraria from MedLink, prIME Oncology, and the American Academic of Neurology (Continuum); and royalties from Wolters Kluwer Health (UpToDate); outside of the submitted work. MMK reports research funding from Blue Earth Diagnostics; outside of the submitted work. MM reports consultant or advisory roles for Zap, Mevion, Karyopharm, Tocagen and Astra-Zeneca; and Board of Directors options from Oncoceutics; outside of the submitted work. MK reports consultant or advisory roles for Janssen, AbbVie, Ipsen, Pfizer Roche, and Jackson Laboratory for Genomic Medicine; and research funding from AbbVie, Bristol-Myers Squibb, and Specialized Therapeutics; outside of the submitted work.

## Publisher’s Note

All claims expressed in this article are solely those of the authors and do not necessarily represent those of their affiliated organizations, or those of the publisher, the editors and the reviewers. Any product that may be evaluated in this article, or claim that may be made by its manufacturer, is not guaranteed or endorsed by the publisher.
