# Supplementary material for: Clinical Trial Eligibility Criteria and Recently Approved Cancer Therapies for Patients With Brain Metastases
Source: Front Oncol. 2022 Jan 3;11:780379. doi: 10.3389/fonc.2021.780379 (PMC8761732; doi:10.3389/fonc.2021.780379)
Supplement: Supplementary file 1 [file Table_1.docx]

**Supplementary Tables**

Supplementary Table 1. Novel FDA approved therapies for cancer between 2018-2020

| Year | Cancer Therapy | Target or Mechanism of Action | Indication | Registrational Trial(s) | Reference |
| --- | --- | --- | --- | --- | --- |
| 2018 | Apalutamide | Androgen receptor inhibitor | Prostate cancer | SPARTAN | (1) |
| 2018 | Binimetinib | MEK inhibitor | *BRAF*-mutated melanoma | COLUMBUS | (2) |
| 2018 | Cemiplimab | PD-1 antibody | Cutaneous squamous cell carcinoma | Study 1423; Study 1540 | (3) |
| 2018 | Dacomitinib | EGFR inhibitor | *EGFR*-mutated non-small cell lung cancer (NSCLC) | ARCHER 1050 | (4) |
| 2018 | Encorafenib | BRAF inhibitor | *BRAF*-mutated melanoma | COLUMBUS | (2) |
| 2018 | Larotrectinib | TRKA, TRKB and TRKC inhibitor | *NTRK*-positive solid cancers | LOXO-TRK-14001; SCOUT; NAVIGATE | (5) |
| 2018 | Lorlatinib | ALK and ROS1 inhibitor | *ALK*-positive NSCLC | Study B7461001 | (6) |
| 2018 | Lutetium Lu 177 dotatate | Somatostatin receptor-targeted radiopharmaceutical | Gastroenterohepatic-neuroendocrine tumours (GEP-NET) | NETTER-1 | (7) |
| 2018 | Talazoparib | PARP inhibitor | *BRCA*-mutated *HER2*-negative breast cancer | EMBRACA | (8) |
| 2018 | Trastuzumab deruxtecan | HER2-directed antibody-drug conjugate (ADC) | *HER2*-positive breast cancer | DESTINY-Breast01 | (9) |
| 2019 | Alpelisib | PI3K inhibitor | Breast cancer | SOLAR-1 | (10) |
| 2019 | Darolutamide | Androgen receptor inhibitor | Prostate cancer | ARAMIS | (11) |
| 2019 | Enfortumab vedotin | Nectin-4-directed ADC | Urothelial cancers | EV-201 | (12) |
| 2019 | Entrectinib | TRKA, TRKB, TRKC, ROS1 and ALK inhibitor | *NTRK*-positive solid tumours and *ROS1*-positive NSCLC | ALKA; STARTRK-1; STARTRK-2 | (13,14) |
| 2019 | Erdafitinib | FGFR inhibitor | Bladder cancer | BLC2001 | (15) |
| 2019 | Pexidartinib | CSF1R, KIT and FLT3 inhibitor | Tenosynovial giant cell tumour | ENLIVEN | (16) |
| 2019 | Zanubrutinib | BTK inhibitor | Mantle cell lymphoma | BGB-3111-206 | (17) |
| 2020 | Avapritinib | PDGFRA and KIT inhibitor | Gastrointestinal stromal tumours with *PDGFRA* exon 18 mutations | NAVIGATOR | (18) |
| 2020 | Capmatinib | MET inhibitor | *MET* exon 14 altered NSCLC | GEOMETRY mono-1 | (19) |
| 2020 | Lurbinectedin | Alkylating chemotherapy | Small-cell lung cancer | Study B-005 | (20) |
| 2020 | Margetuximab | HER2-directed antibody | *HER2*-positive breast cancer | SOPHIA | (21) |
| 2020 | Pemigatinib | FGFR1-3 inhibitor | Cholangiocarcinoma | FIGHT-202 | (22) |
| 2020 | Pralsetinib | RET inhibitor | *RET* fusion-positive NSCLC | ARROW | (23) |
| 2020 | Relugolix | GnRH receptor antagonist | Prostate cancer | HERO | (24) |
| 2020 | Ripretinib | KIT and PDGFRA inhibitor | Gastrointestinal stromal tumour | INVICTUS | (25) |
| 2020 | Sacituzumab govitecan | TROP2-directed ADC, with topoisomerase inhibitor | Triple-negative breast cancer | IMMU-132-01 | (26) |
| 2020 | Selpercatinib | RET inhibitor | *RET* fusion-positive NSCLC and thyroid cancer | LIBRETTO-001 | (27,28) |
| 2020 | Tafasitamab | CD19-directed mAb | Diffuse large B cell lymphoma | L-MIND | (29) |
| 2020 | Tazemetostat | EZH2 inhibitor | Epithelioid sarcoma | Study EZH-202 | (30) |
| 2020 | Tucatinib | HER2 inhibitor | *HER2*-positive breast cancer | HER2CLIMB | (31) |

Supplementary Table 2. Registrational trial characteristics for newly FDA approved cancer therapies from 2018-2020

| Characteristic | N (%) |
| --- | --- |
| Drug property  Antibody-drug conjugate  Chemotherapy  Hormonal therapy  Monoclonal antibody (immunotherapy)  Monoclonal antibody (targeted)  Radiopharmaceutical  Small molecule inhibitor | 3 (10)  1 (3)  3 (10)  1 (3)  2 (7)  1 (3)  19 (63) |
| Registrational trial phase  1  1/2  2  3 | 1 (3)  6 (20)  10 (33)  13 (43) |
| Disease setting  Early-stage, locally advanced or non-metastatic  Metastatic, advanced, or late stage | 3 (10)  27 (90) |

**References**

1. Smith MR, Saad F, Chowdhury S, Oudard S, Hadaschik BA, Graff JN, Olmos D, Mainwaring PN, Lee JY, Uemura H, et al. Apalutamide Treatment and Metastasis-free Survival in Prostate Cancer. *N Engl J Med* (2018) **378**:1408–1418. doi: 10.1056/NEJMoa1715546

2. Dummer R, Ascierto PA, Gogas HJ, Arance A, Mandala M, Liszkay G, Garbe C, Schadendorf D, Krajsova I, Gutzmer R, et al. Encorafenib plus binimetinib versus vemurafenib or encorafenib in patients with BRAF-mutant melanoma (COLUMBUS): a multicentre, open-label, randomised phase 3 trial. *Lancet Oncol* (2018) **19**:603–615. doi: 10.1016/S1470-2045(18)30142-6

3. Migden MR, Rischin D, Schmults CD, Guminski A, Hauschild A, Lewis KD, Chung CH, Hernandez-Aya L, Lim AM, Chang ALS, et al. PD-1 Blockade with Cemiplimab in Advanced Cutaneous Squamous-Cell Carcinoma. *N Engl J Med* (2018) **379**:341–351. doi: 10.1056/NEJMoa1805131

4. Wu Y-L, Cheng Y, Zhou X, Lee KH, Nakagawa K, Niho S, Tsuji F, Linke R, Rosell R, Corral J, et al. Dacomitinib versus gefitinib as first-line treatment for patients with EGFR-mutation-positive non-small-cell lung cancer (ARCHER 1050): a randomised, open-label, phase 3 trial. *Lancet Oncol* (2017) **18**:1454–1466. doi: 10.1016/S1470-2045(17)30608-3

5. Hong DS, DuBois SG, Kummar S, Farago AF, Albert CM, Rohrberg KS, van Tilburg CM, Nagasubramanian R, Berlin JD, Federman N, et al. Larotrectinib in patients with TRK fusion-positive solid tumours: a pooled analysis of three phase 1/2 clinical trials. *Lancet Oncol* (2020) **21**:531–540. doi: 10.1016/S1470-2045(19)30856-3

6. Solomon BJ, Besse B, Bauer TM, Felip E, Soo RA, Camidge DR, Chiari R, Bearz A, Lin C-C, Gadgeel SM, et al. Lorlatinib in patients with ALK-positive non-small-cell lung cancer: results from a global phase 2 study. *Lancet Oncol* (2018) **19**:1654–1667. doi: 10.1016/S1470-2045(18)30649-1

7. Strosberg J, El-Haddad G, Wolin E, Hendifar A, Yao J, Chasen B, Mittra E, Kunz PL, Kulke MH, Jacene H, et al. Phase 3 Trial of 177Lu-Dotatate for Midgut Neuroendocrine Tumors. *N Engl J Med* (2017) **376**:125–135. doi: 10.1056/NEJMoa1607427

8. Litton JK, Rugo HS, Ettl J, Hurvitz SA, Gonçalves A, Lee K-H, Fehrenbacher L, Yerushalmi R, Mina LA, Martin M, et al. Talazoparib in Patients with Advanced Breast Cancer and a Germline BRCA Mutation. *N Engl J Med* (2018) **379**:753–763. doi: 10.1056/NEJMoa1802905

9. Modi S, Saura C, Yamashita T, Park YH, Kim S-B, Tamura K, Andre F, Iwata H, Ito Y, Tsurutani J, et al. Trastuzumab Deruxtecan in Previously Treated HER2-Positive Breast Cancer. *N Engl J Med* (2020) **382**:610–621. doi: 10.1056/NEJMoa1914510

10. André F, Ciruelos E, Rubovszky G, Campone M, Loibl S, Rugo HS, Iwata H, Conte P, Mayer IA, Kaufman B, et al. Alpelisib for PIK3CA-Mutated, Hormone Receptor-Positive Advanced Breast Cancer. *N Engl J Med* (2019) **380**:1929–1940. doi: 10.1056/NEJMoa1813904

11. Fizazi K, Shore N, Tammela TL, Ulys A, Vjaters E, Polyakov S, Jievaltas M, Luz M, Alekseev B, Kuss I, et al. Darolutamide in Nonmetastatic, Castration-Resistant Prostate Cancer. *N Engl J Med* (2019) **380**:1235–1246. doi: 10.1056/NEJMoa1815671

12. Rosenberg JE, O’Donnell PH, Balar AV, McGregor BA, Heath EI, Yu EY, Galsky MD, Hahn NM, Gartner EM, Pinelli JM, et al. Pivotal Trial of Enfortumab Vedotin in Urothelial Carcinoma After Platinum and Anti-Programmed Death 1/Programmed Death Ligand 1 Therapy. *J Clin Oncol* (2019) **37**:2592–2600. doi: 10.1200/JCO.19.01140

13. Drilon A, Siena S, Dziadziuszko R, Barlesi F, Krebs MG, Shaw AT, de Braud F, Rolfo C, Ahn M-J, Wolf J, et al. Entrectinib in ROS1 fusion-positive non-small-cell lung cancer: integrated analysis of three phase 1-2 trials. *Lancet Oncol* (2020) **21**:261–270. doi: 10.1016/S1470-2045(19)30690-4

14. Doebele RC, Drilon A, Paz-Ares L, Siena S, Shaw AT, Farago AF, Blakely CM, Seto T, Cho BC, Tosi D, et al. Entrectinib in patients with advanced or metastatic NTRK fusion-positive solid tumours: integrated analysis of three phase 1-2 trials. *Lancet Oncol* (2020) **21**:271–282. doi: 10.1016/S1470-2045(19)30691-6

15. Loriot Y, Necchi A, Park SH, Garcia-Donas J, Huddart R, Burgess E, Fleming M, Rezazadeh A, Mellado B, Varlamov S, et al. Erdafitinib in Locally Advanced or Metastatic Urothelial Carcinoma. *N Engl J Med* (2019) **381**:338–348. doi: 10.1056/NEJMoa1817323

16. Tap WD, Gelderblom H, Palmerini E, Desai J, Bauer S, Blay J-Y, Alcindor T, Ganjoo K, Martín-Broto J, Ryan CW, et al. Pexidartinib versus placebo for advanced tenosynovial giant cell tumour (ENLIVEN): a randomised phase 3 trial. *Lancet* (2019) **394**:478–487. doi: 10.1016/S0140-6736(19)30764-0

17. Song Y, Zhou K, Zou D, Zhou J, Hu J, Yang H, Zhang H, Ji J, Xu W, Jin J, et al. Treatment of Patients with Relapsed or Refractory Mantle-Cell Lymphoma with Zanubrutinib, a Selective Inhibitor of Bruton’s Tyrosine Kinase. *Clin Cancer Res* (2020) **26**:4216–4224. doi: 10.1158/1078-0432.CCR-19-3703

18. Heinrich MC, Jones RL, von Mehren M, Schöffski P, Serrano C, Kang Y-K, Cassier PA, Mir O, Eskens F, Tap WD, et al. Avapritinib in advanced PDGFRA D842V-mutant gastrointestinal stromal tumour (NAVIGATOR): a multicentre, open-label, phase 1 trial. *Lancet Oncol* (2020) **21**:935–946. doi: 10.1016/S1470-2045(20)30269-2

19. Wolf J, Seto T, Han J-Y, Reguart N, Garon EB, Groen HJM, Tan DSW, Hida T, de Jonge M, Orlov SV, et al. Capmatinib in MET Exon 14-Mutated or MET-Amplified Non-Small-Cell Lung Cancer. *N Engl J Med* (2020) **383**:944–957. doi: 10.1056/NEJMoa2002787

20. Trigo J, Subbiah V, Besse B, Moreno V, López R, Sala MA, Peters S, Ponce S, Fernández C, Alfaro V, et al. Lurbinectedin as second-line treatment for patients with small-cell lung cancer: a single-arm, open-label, phase 2 basket trial. *Lancet Oncol* (2020) **21**:645–654. doi: 10.1016/S1470-2045(20)30068-1

21. Rugo HS, Im S-A, Cardoso F, Cortés J, Curigliano G, Musolino A, Pegram MD, Wright GS, Saura C, Escrivá-de-Romaní S, et al. Efficacy of Margetuximab vs Trastuzumab in Patients With Pretreated ERBB2-Positive Advanced Breast Cancer: A Phase 3 Randomized Clinical Trial. *JAMA Oncol* (2021) **7**:573–584. doi: 10.1001/jamaoncol.2020.7932

22. Abou-Alfa GK, Sahai V, Hollebecque A, Vaccaro G, Melisi D, Al-Rajabi R, Paulson AS, Borad MJ, Gallinson D, Murphy AG, et al. Pemigatinib for previously treated, locally advanced or metastatic cholangiocarcinoma: a multicentre, open-label, phase 2 study. *Lancet Oncol* (2020) **21**:671–684. doi: 10.1016/S1470-2045(20)30109-1

23. Gainor JF, Curigliano G, Kim D-W, Lee DH, Besse B, Baik CS, Doebele RC, Cassier PA, Lopes G, Tan DSW, et al. Pralsetinib for RET fusion-positive non-small-cell lung cancer (ARROW): a multi-cohort, open-label, phase 1/2 study. *Lancet Oncol* (2021) **22**:959-69. doi: 10.1016/S1470-2045(21)00247-3

24. Shore ND, Saad F, Cookson MS, George DJ, Saltzstein DR, Tutrone R, Akaza H, Bossi A, van Veenhuyzen DF, Selby B, et al. Oral Relugolix for Androgen-Deprivation Therapy in Advanced Prostate Cancer. *N Engl J Med* (2020) **382**:2187–2196. doi: 10.1056/NEJMoa2004325

25. Blay J-Y, Serrano C, Heinrich MC, Zalcberg J, Bauer S, Gelderblom H, Schöffski P, Jones RL, Attia S, D’Amato G, et al. Ripretinib in patients with advanced gastrointestinal stromal tumours (INVICTUS): a double-blind, randomised, placebo-controlled, phase 3 trial. *Lancet Oncol* (2020) **21**:923–934. doi: 10.1016/S1470-2045(20)30168-6

26. Bardia A, Mayer IA, Vahdat LT, Tolaney SM, Isakoff SJ, Diamond JR, O’Shaughnessy J, Moroose RL, Santin AD, Abramson VG, et al. Sacituzumab Govitecan-hziy in Refractory Metastatic Triple-Negative Breast Cancer. *N Engl J Med* (2019) **380**:741–751. doi: 10.1056/NEJMoa1814213

27. Drilon A, Oxnard GR, Tan DSW, Loong HHF, Johnson M, Gainor J, McCoach CE, Gautschi O, Besse B, Cho BC, et al. Efficacy of Selpercatinib in RET Fusion-Positive Non-Small-Cell Lung Cancer. *N Engl J Med* (2020) **383**:813–824. doi: 10.1056/NEJMoa2005653

28. Wirth LJ, Sherman E, Robinson B, Solomon B, Kang H, Lorch J, Worden F, Brose M, Patel J, Leboulleux S, et al. Efficacy of Selpercatinib in RET-Altered Thyroid Cancers. *N Engl J Med* (2020) **383**:825–835. doi: 10.1056/NEJMoa2005651

29. Salles G, Duell J, González Barca E, Tournilhac O, Jurczak W, Liberati AM, Nagy Z, Obr A, Gaidano G, André M, et al. Tafasitamab plus lenalidomide in relapsed or refractory diffuse large B-cell lymphoma (L-MIND): a multicentre, prospective, single-arm, phase 2 study. *Lancet Oncol* (2020) **21**:978–988. doi: 10.1016/S1470-2045(20)30225-4

30. Gounder M, Schöffski P, Jones RL, Agulnik M, Cote GM, Villalobos VM, Attia S, Chugh R, Chen TW-W, Jahan T, et al. Tazemetostat in advanced epithelioid sarcoma with loss of INI1/SMARCB1: an international, open-label, phase 2 basket study. *Lancet Oncol* (2020) **21**:1423–1432. doi: 10.1016/S1470-2045(20)30451-4

31. Murthy RK, Loi S, Okines A, Paplomata E, Hamilton E, Hurvitz SA, Lin NU, Borges V, Abramson V, Anders C, et al. Tucatinib, Trastuzumab, and Capecitabine for HER2-Positive Metastatic Breast Cancer. *N Engl J Med* (2020) **382**:597–609. doi: 10.1056/NEJMoa1914609
